# Supplementary figures and images for: TAK-071, a muscarinic M1 receptor positive allosteric modulator, attenuates scopolamine-induced quantitative electroencephalogram power spectral changes in cynomolgus monkeys
Source: PLoS One. 2019 Mar 11;14(3):e0207969. doi: 10.1371/journal.pone.0207969 (PMC6411103; doi:10.1371/journal.pone.0207969)

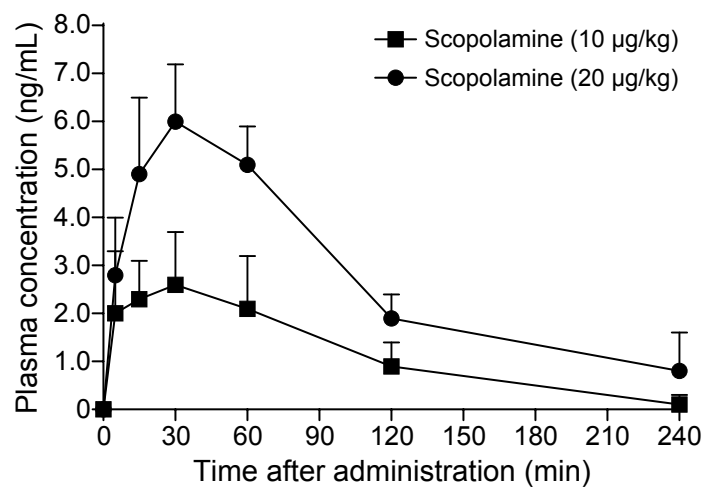

Supplement: S1 Fig — Scopolamine (10 or 20 μg/kg) was administered subcutaneously to cynomolgus monkeys. After treatment with scopolamine, plasma samples were collected at 5, 15, 30, 60, 120 and 240 min. Results represent mean ± SD for 3 monkeys in each group. SD, standard deviation. (PDF) [file pone.0207969.s001.pdf]

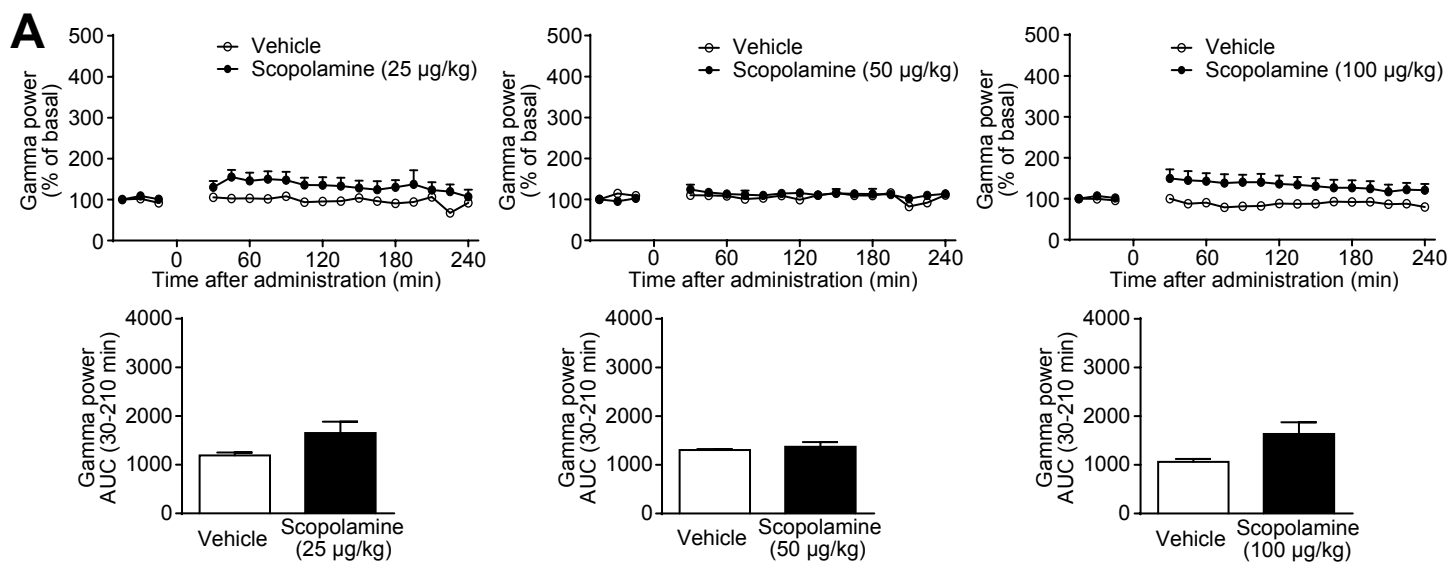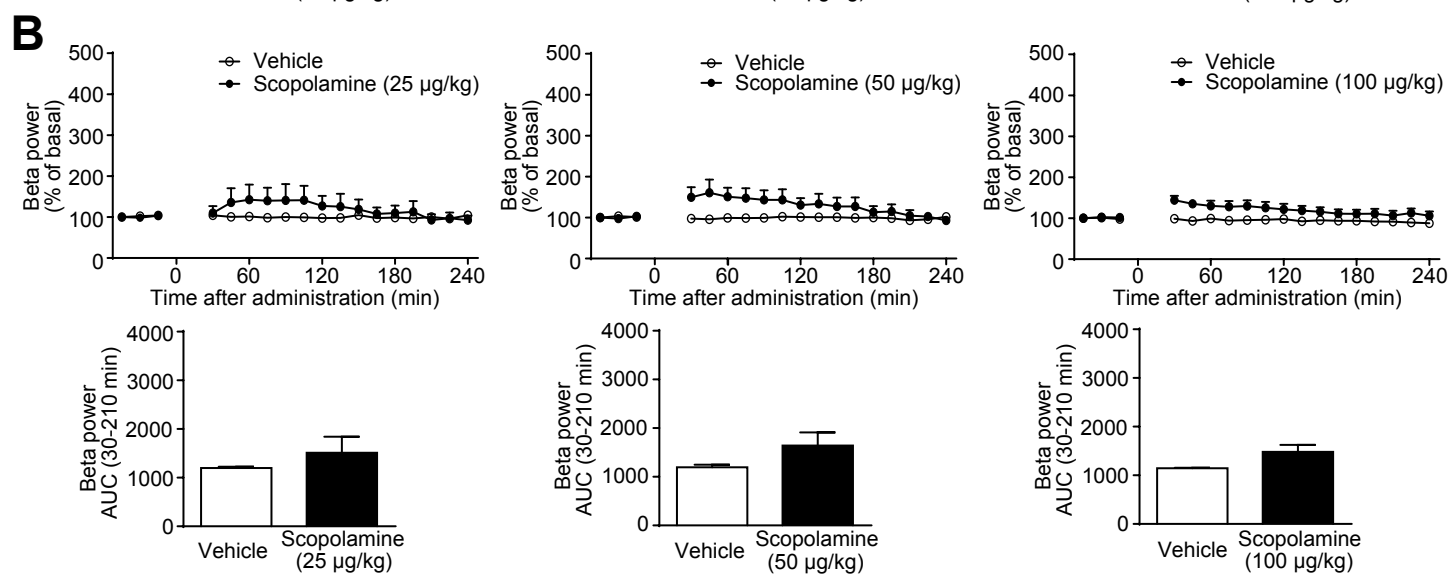

Supplement: S2 Fig — Scopolamine (25–100 μg/kg) was administered subcutaneously to cynomolgus monkeys. After treatment with scopolamine or vehicle, (A) gamma and (B) beta power bands of qEEG spectra were measured from 30 to 240 min. Results represent mean ± SEM of AUC between 30 and 210 min after treatment for 4 monkeys in each group. AUC, area under the curve; qEEG, quantitative electroencephalogram; SEM, standard error of the mean. (PDF) [file pone.0207969.s002.pdf]

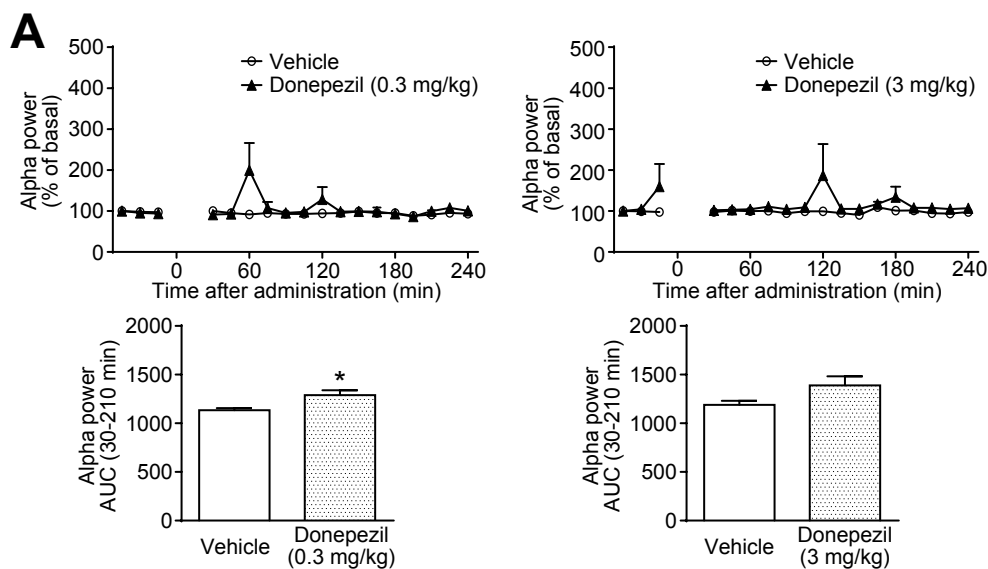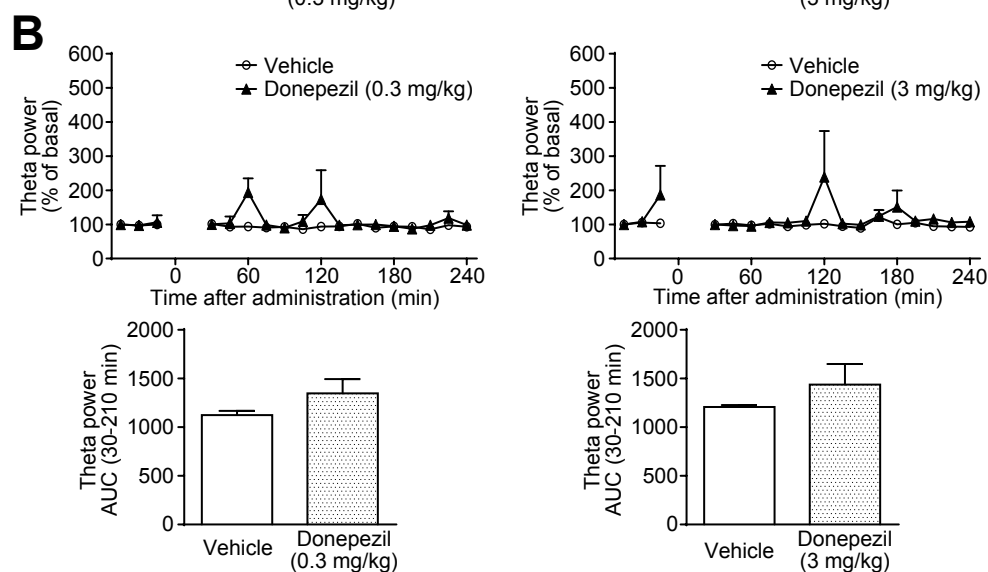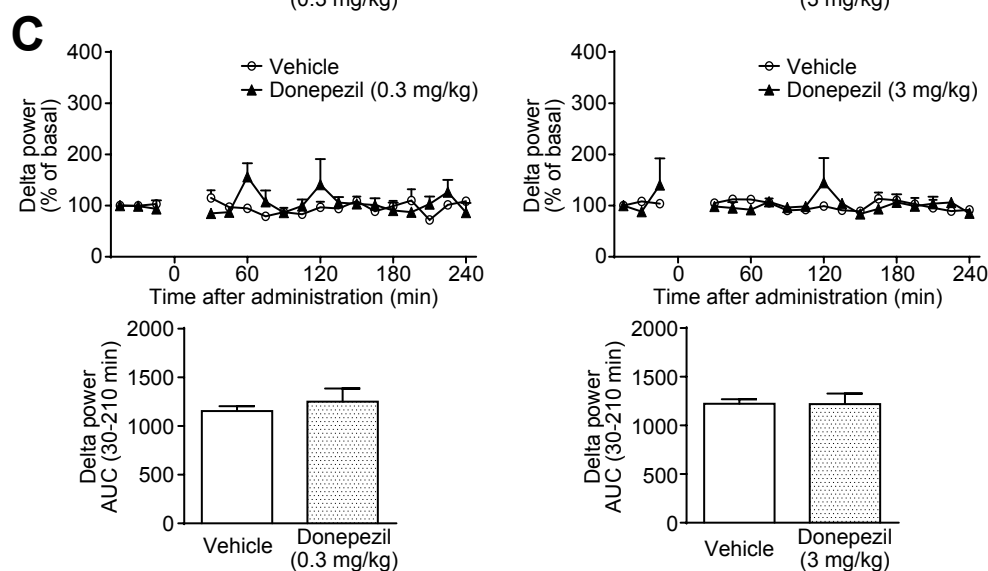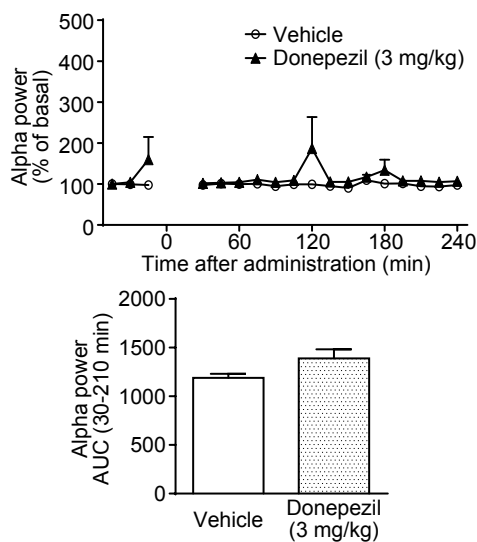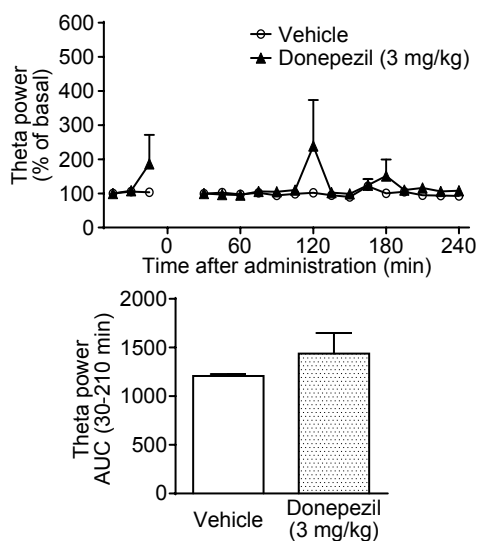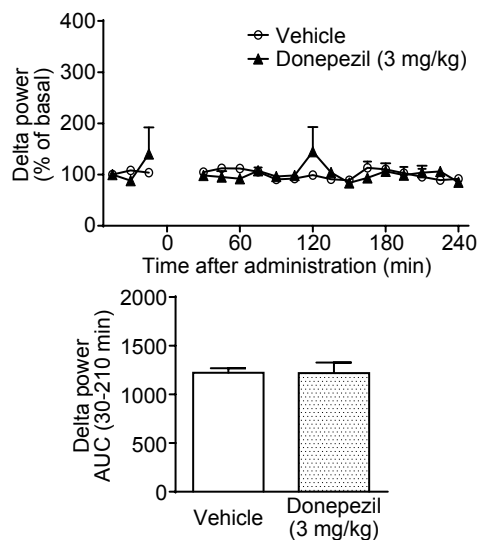

Supplement: S3 Fig — Donepezil (0.3 or 3 mg/kg, p.o.) was administered to cynomolgus monkeys. After treatment with donepezil or vehicle, (A) alpha, (B) theta, and (C) delta power bands of qEEG spectra were measured from 30 to 240 min. Results represent mean ± SEM of AUC between 30 and 210 min after treatment for 3–4 monkeys in each group. *P ≤ 0.05 versus vehicle-treated group by paired t-test. AUC, area under the curve; p.o., oral administration; qEEG, quantitative electroencephalogram; SEM, standard error of the mean. (PDF) [file pone.0207969.s003.pdf]

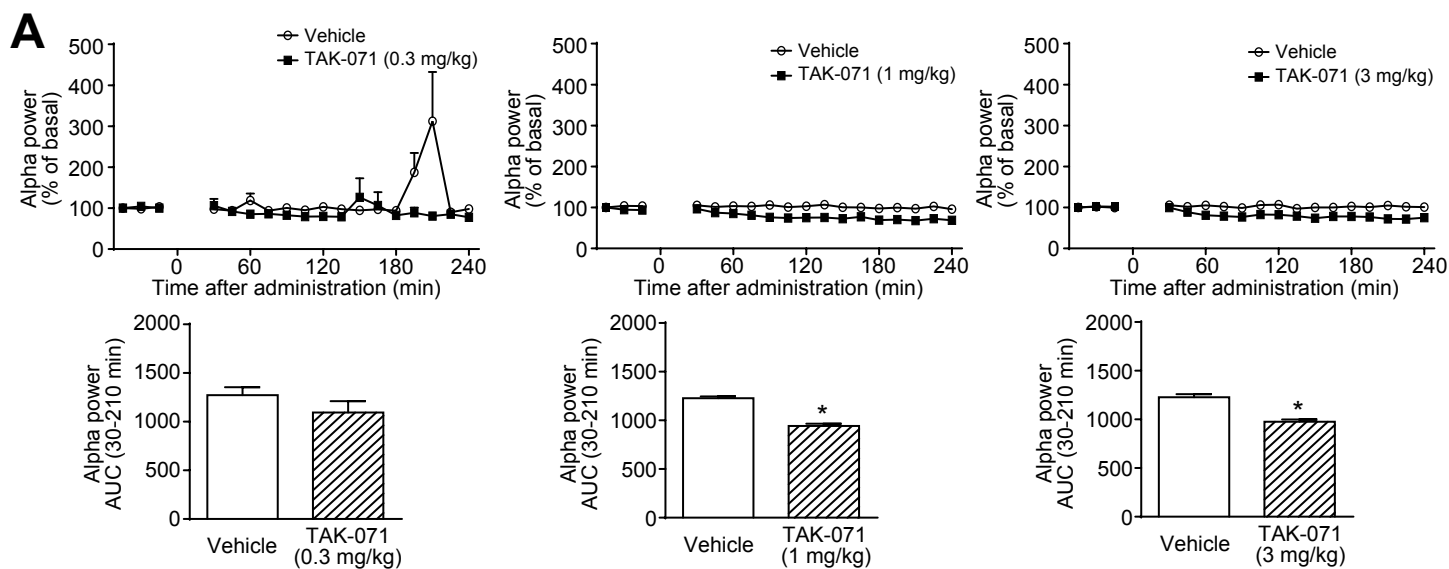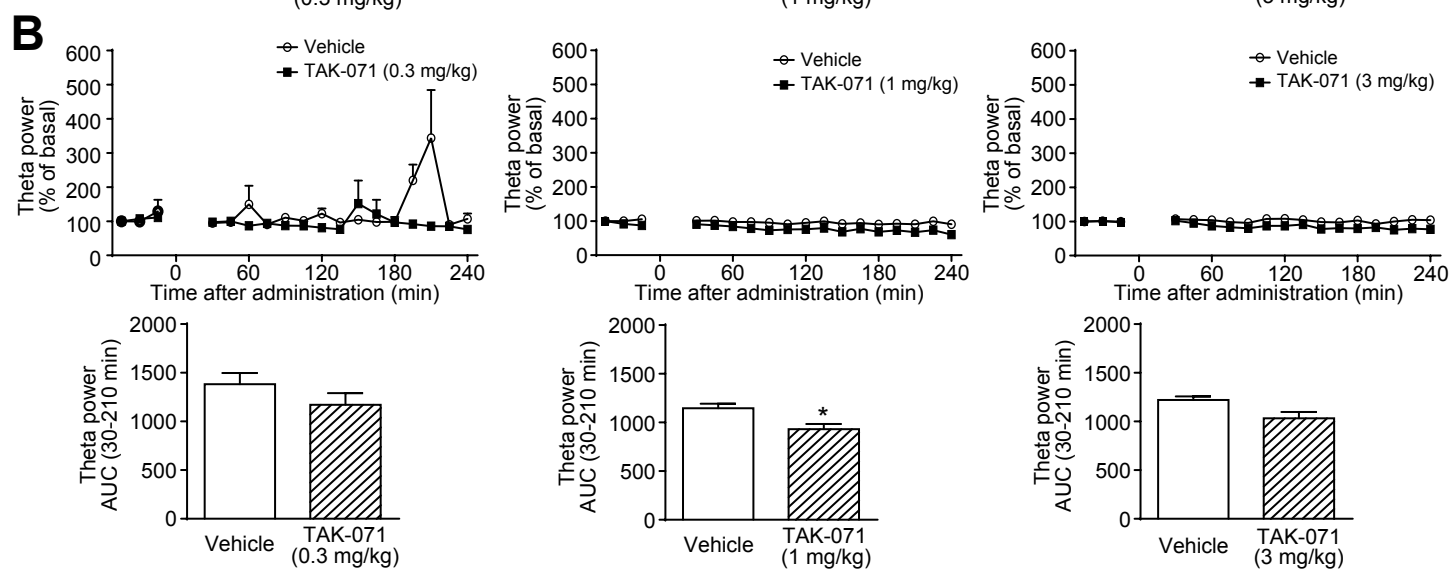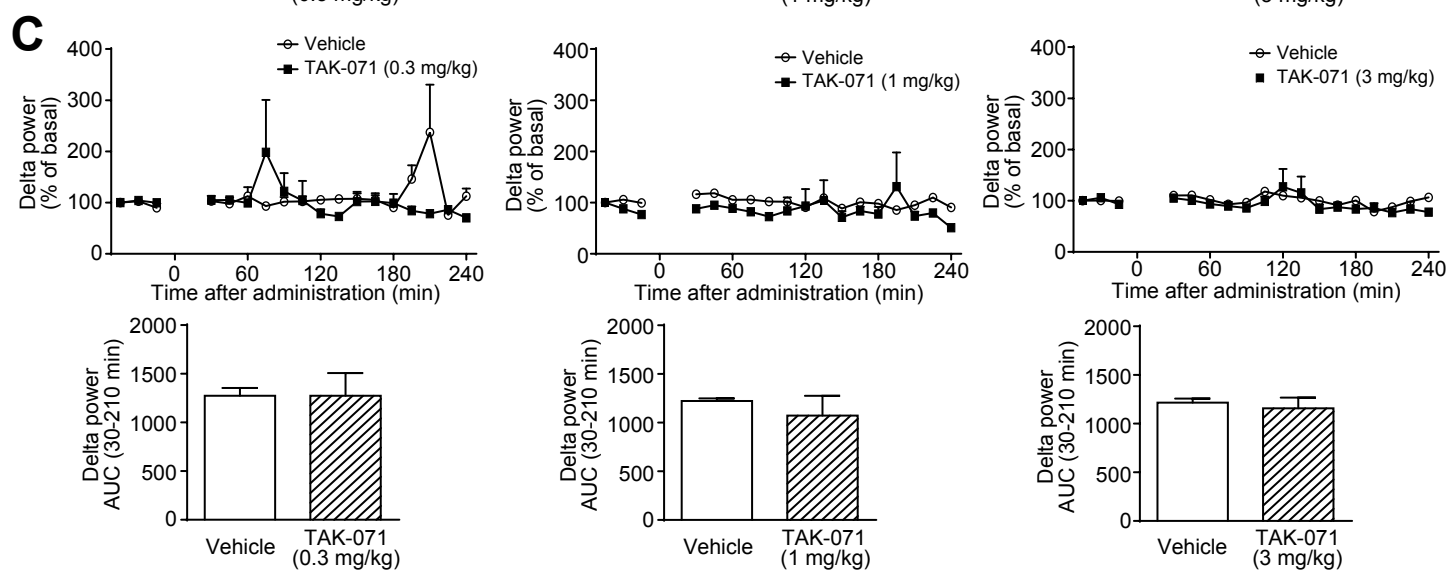

Supplement: S4 Fig — TAK-071 (0.3–3 mg/kg, p.o.) was administered to cynomolgus monkeys. After treatment with TAK-071 or vehicle, (A) alpha, (B) theta, and (C) delta power bands of qEEG spectra were measured from 30 to 240 min. Results represent mean ± SEM of AUC between 30 and 210 min after treatment for 4 monkeys in each group. *P ≤ 0.05 versus vehicle-treated group by paired t-test. AUC, area under the curve; p.o., oral administration; qEEG, quantitative electroencephalogram; SEM, standard error of the mean. (PDF) [file pone.0207969.s004.pdf]
