# Supplementary material for: TAK-071, a muscarinic M1 receptor positive allosteric modulator, attenuates scopolamine-induced quantitative electroencephalogram power spectral changes in cynomolgus monkeys
Source: PLoS One. 2019 Mar 11;14(3):e0207969. doi: 10.1371/journal.pone.0207969 (PMC6411103; doi:10.1371/journal.pone.0207969)
Supplement: S1 Table — Scopolamine (10 or 20 μg/kg) was administered subcutaneously to cynomolgus monkeys. After treatment with scopolamine, plasma sample was collected at 5, 15, 30, 60, 120, and 240 min. Cmax, Tmax and AUC0–4h were calculated from the results of S2 Fig. Results represent mean ± SD for 3 monkeys in each group. (PDF) [file pone.0207969.s005.pdf]

PK parameter	Scopolamine HBr	
	10 µg/kg	20 µg/kg
**Cmax (ng/mL)**	2.8 ± 1.2	6.1 ± 1.1
**Tmax (h)**	0.3 ± 0.2	0.7 ± 0.3
**AUC_{0-4h} (ng·h/mL)**	4.7 ± 2.3	11.0 ± 2.1
