# Supplementary material for: TAK-071, a muscarinic M1 receptor positive allosteric modulator, attenuates scopolamine-induced quantitative electroencephalogram power spectral changes in cynomolgus monkeys
Source: PLoS One. 2019 Mar 11;14(3):e0207969. doi: 10.1371/journal.pone.0207969 (PMC6411103; doi:10.1371/journal.pone.0207969)
Supplement: S2 Table — TAK-071 (0.1 or 1 mg/kg) was orally administered to cynomolgus monkeys. After treatment with TAK-071, plasma sample was collected at 15, 30 min, and 1, 2, 4, 6, 8, 24, 48, and 72 hr. Cmax, Tmax and AUC0–72h were determined. Results represent mean ± SD for 3 monkeys in each group. (PDF) [file pone.0207969.s006.pdf]

**PK parameter**	**TAK-071**	
	0.1 mg/kg	1 mg/kg
**Cmax (ng/mL)**	97.9 ± 16.8	833 ± 75
**Tmax (h)**	3.3 ± 2.3	4.7 ± 1.2
**$AUC_{0\text{-}72h}$ (ng·h/mL)**	1710 ± 240	16700 ± 1700
